# Review of different methods used for clinical recognition and assessment of pain in dogs and cats

**DOI:** 10.1080/23144599.2019.1680044

**Published:** 2019-11-18

**Authors:** Ismael Hernandez-Avalos, Daniel Mota-Rojas, Patricia Mora-Medina, Julio Martínez-Burnes, Alejandro Casas Alvarado, Antonio Verduzco-Mendoza, Karina Lezama-García, Adriana Olmos-Hernandez

**Affiliations:** a Departament of Biological Science, FESC, Universidad Nacional Autónoma de México (UNAM); b Neurophysiology of Pain, Behavior and Assessment of Welfare in Domestic Animals, DPAA, Universidad Autónoma Metropolitana (UAM), Mexico City, Mexico; c Livestock Science Department, Universidad Nacional Autónoma de México (UNAM), FESC, Mexico; d Graduate and Research Department, Facultad de Medicina Veterinaria y Zootecnia, Universidad Autónoma de Tamaulipas, Victoria City, Tamaulipas, Mexico; e Departamento Bioterio y Cirugía Experimental, Instituto Nacional de Rehabilitación-Luis Guillermo Ibarra Ibarra (INR-LGII), Mexico City, Mexico

**Keywords:** Analgesia, acute pain, animal welfare, bispectral index, dogs, cats

## Abstract

In light of the need to perform surgical techniques and the importance of animal welfare because of acute pain, the objectives of the veterinary anaesthetists are to manage muscle relaxation and adequate analgesia in order to conserve a balance in the autonomic nervous system, enhance the action of the parasympathetic system in the face of the emerging action of the sympathetic portion provoked by the surgeon, and maintain a balance among them. The aim of the present review is to describe different evaluation criteria for acute pain using unidimensional and multidimensional scales, correlating these findings to parasympathetic tone activity (PTA) and bispectral index (BIS) assessment, to conduct an objective evaluation of pain that patients (dog or cat) perceives, in order to administrate an adequate analgesic treatment in each case. In conclusion, this integral, objective evaluation will allow veterinarians – especially anaesthesiologists – to improve the management of pain in the patients.

## 1. Introduction

As a part of standard medical practice, veterinarians must include pain and suffer relieve for their patients [1], having as a first step the recognizing of this state to achieve a correct diagnosis and prescribe adequate analgesic treatment, not only for ethical reasons related to animal welfare but also to ensure proper medical and surgical procedures. Therefore, pain management is currently an integrated medical and surgical aspect in veterinary medicine [2,3], making necessary the use of objective parameters to quantify the severity of the pain experienced [4,5].

Efforts to unify the criteria for measuring pain have led to the development of different guides used to recognize this condition based on physical and biological alterations such as behavioural changes [1], facial expression [6], physiological parameters, and biochemical mediators [7,8]. However, these have not proven to be effective because just one parameter can not apply to all animal species since this will differ from one species to another, and even among individuals of the same species [4,9].

Concerning surgical stimuli, the main objective is to maintain adequate intraoperative analgesia to prevent the risks that can affect patient recovery [9–11]. For this reason, specialized equipment and protocols to measure acute pain have been developed [12–14], leading to a better knowledge of nociceptive pathways and mechanisms of action that can help veterinarians to deal with the pain before it appears (preventive analgesia), since inadequately pain treatment causes unnecessary suffering, predisposes patients to medical complications, significantly increases hospital stays, extends recovery time and increases the cost of surgical procedures. For all these reasons, pain must be prevented through optimal pharmacological and/or physical treatments that can act at different levels according to the point of origin of the pain [15,16].

Because nociception compromises animal welfare and that it is challenging to homologate the criteria for its assessment in all animal species, the purpose of this paper the purpose of this paper is to review the different different methods documented and validated for the qualitative and quantitative measurement of pain in dogs and cats.

### 1.1. The neurobiology of acute pain

Due to the complexity of assessing pain and the multitude of aspects and variants in the animal behaviour when they are under pain [1,17], it is now believed that the neuronal process through which potentially harmful stimuli to tissues are encoded and processed (nociception is a multidimensional experience that includes somatosensory, cognitive and emotional components) [18,19]. Recently the most widely-accepted definition is the one published by the International Association for the Study of Pain (IASP), which defines this condition as: “an unpleasant sensory or emotional experience associated with real or potential tissue damage that is described in terms of that damage” [20,21]. In this sense, pain is a subjective and ineffable concept that exists as long as individuals manifest that something “hurts” during an evaluation [22].

The pathophysiological mechanisms of nociception (i.e., the neurophysiological process of pain) are similar among mammals, which can suffer as sensitive creatures. Moreover, observations show that both animals and humans develop the same neuronal process of recognizing, conducting and modulating pain [23–26].

**Figure 1.**
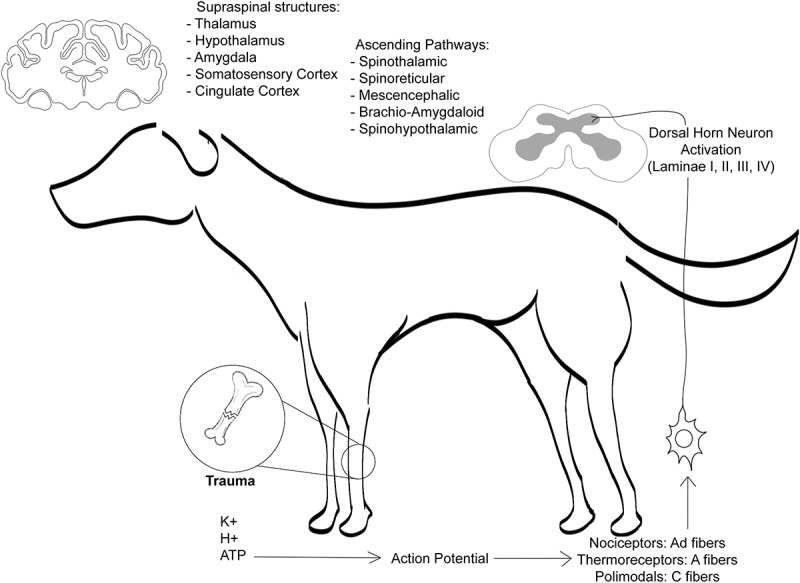
The nociceptive pathway transmits, modulates and integrates signals at different levels of the nervous system, from peripheral nociceptors to higher brain centres (thalamus and cortex). Own elaboration, using information from Stafford [32]; Lemke [33].

Like all sensory and alarm systems, nociception contains the mechanisms required for the sensation of pain to be received at some level of the periphery (skin, viscera, skeletal muscle) and then be conducted to the central nervous system (CNS), where it is processed and consciously integrated (at the spinal and supraspinal levels) [27,28]. During this process, the peripheral responses to tissue damage include the local release of substances like histamine, prostaglandins, hydrogen ions and potassium, which are known as the “inflammatory soup” [29–31] (Figure 1 [32,33]).

### 1.2. Pain neurophysiology processes

**Figure 2.**
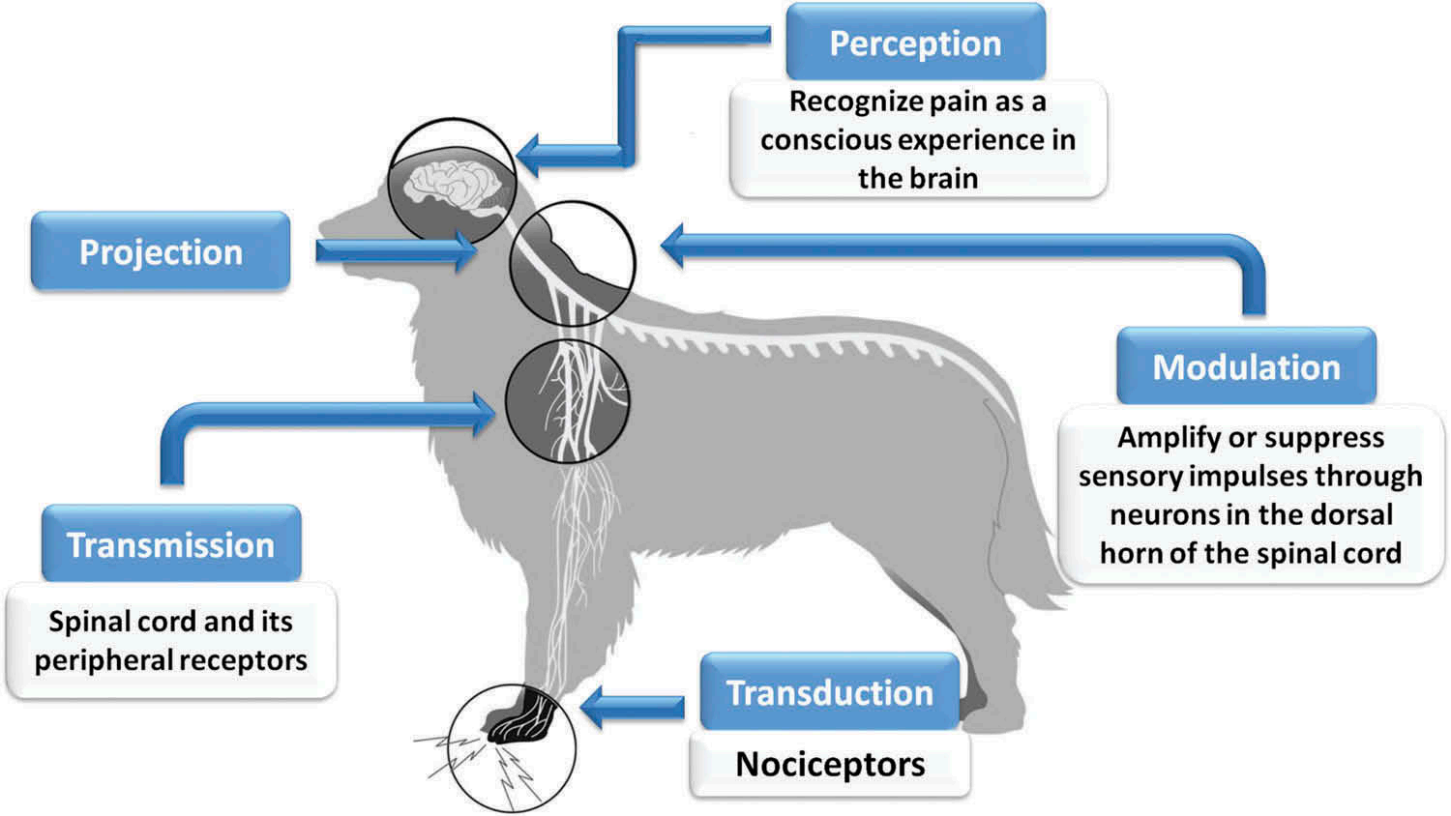
Diagram of the pain pathway. Own elaboration, using information from Lamont [34].

To facilitate the comprehension of the neuro-physiological process of pain is divided into five consecutive processes once the painful or nociceptive stimulus enters in contact with the organism (Figure 2 [34]) [35]. These stages are:

- Transduction: the nociceptive stimulus is transformed into an electrical signal in the receptors (nociceptors) [36], causing peripheral changes that are recognized as indicators of pain, including redness, swelling and translucence of the skin.
- Transmission: is the conduction of the electrical signal generated in the nociceptors through the axons of the first-order neurons, making synapsis with the second-order neurons in the dorsal horn of the spine [37,38]. This information is transmitted through two primary afferent nociceptive neurons, the C fibres or C polymodal nociceptors (which transmit chemical, thermal and mechanical nociceptive information), and the A-delta fibres (which respond to high-intensity mechanical stimuli and thus are known as high-threshold mechanoreceptors).
- Modulation: the process through which the excitatory and inhibitory mechanisms alter the transmission of the nerve impulse [36].
- Projection: the nociceptive information is transported to the brain through the nervous tracts that originate in the dorsal horn, especially spinothalamic and spinoreticular (supraspinal structures) [39].
- Perception: consists in processing and integrating the information that occurs in multiple, specific areas of the brain, such as the thalamus and cerebral cortex, where sensory characteristics are defined, including the onset, location and type of nociceptive stimulus [37].

## 2. Assessing pain

Pain can provoke physiological alterations and maladaptive behaviours, such as restlessness, unease, sleep disturbance, or resistance to seeking repose [40]. Despite the diversity of responses, the clinical signs that occur as reactions to nociceptive stimuli are constant and obvious. Examples of these include somatic responses (abnormal postures) and autonomic responses like increased heartbeat and high blood pressure [4,8,41].

### 2.1. Physiological level

When animals feel pain, the physiological parameters (heart rate, respiratory rate, pupillary diameter and blood pressure) can be altered as an autonomic response to nociception. However, these parameters are affected by many factors, including fear and stress [42,43], so one single physiological parameter is not a reliable tool for assessing pain precisely [39], though some authors recommend taking them as a basis for recognizing this process [23,44]. In the case of animals that suffer chronic pain, however, these variations tend to be less evident [19].

The physiological changes present in animals after a noxious stimulus that authors have described are mydriasis, tachycardia, tachypnoea and arterial hypertension (with increases of 20% or more; but measured from basal levels, not reference values) [45].

Other indicators described include hormonal concentrations and measurement of chemical mediators. Examples of these are changes in plasma hormone concentrations like cortisol, β-endorphins and catecholamines, which have been considered indirect indicators of pain. To date, however, only weak correlations between indicators of behaviour and increases in plasma levels of these hormones have been determined [46]. Furthermore, it is well-known that the relation among physiological stress, behavioural disorders and pain is complex; therefore endocrine measures can reflect stress responses that may not always be related directly to pain or its severity, so they cannot be used as reliable indicators for evaluating nociception [5,47,48].

Similarly, it has been proven that plasma cortisol is not a biomarker specifically associated with pain in animals since it participates as an indicator of the severity of the inflammation of an illness [5,49]. Lactate levels (a biomolecule produced through cellular metabolism) has been analysed in dogs and cats, since they have traditionally been used as parameters to determine the severity of tissue damage after a traumatic event or a severe metabolic sickness that compromises blood perfusion to tissues, using it as an indicator for prognoses. This indicator has been associated with pain since, in some cases, its elevation can depend on catecholamine production [50–52].

### 2.2. Behavioural level

In animals, it is possible to infer a motor response to pain, causing complex behaviours that are unique to each species [53]. For this reason, behavioural changes have been recognized as indicators of pain in animals [54], where this unpleasant emotional and sensory experience generates changes in behaviour, showing indications of the presence, location and severity of pain [5]. According to Wiseman-Orr et al. [55], the behavioural changes that must be considered in animals are aggression, vocalizations, self-mutilation, social interaction, sleep alteration, restlessness, and reluctance to move (lethargy) [40]. While each species manifests its behaviours related to pain or conduct disorders, they are unique and not applicable to other species.

**Figure 3.**
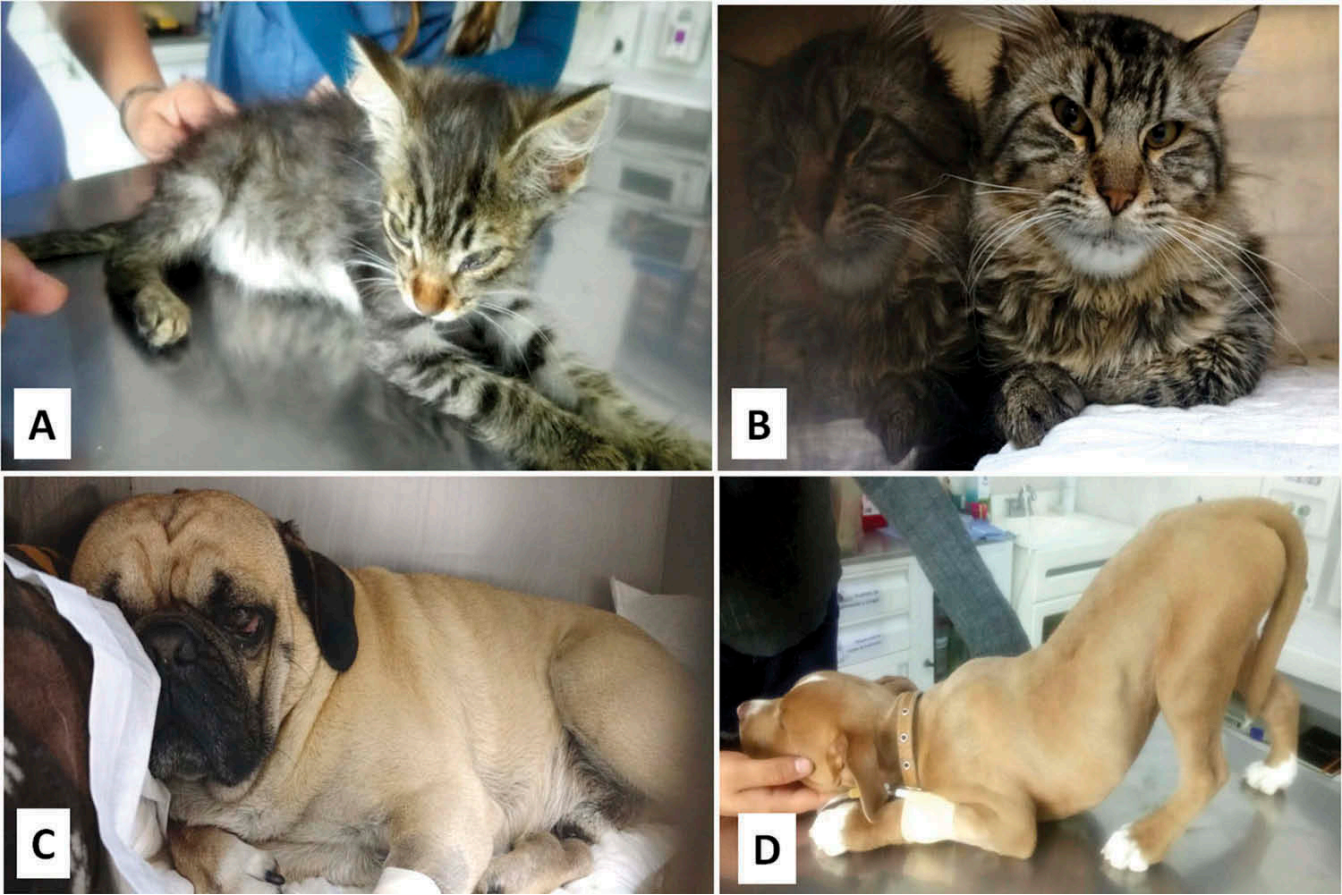
(a) Third eyelid protusion, miosis and messy coat are clinical signs of pain in cats. (b) Avoiding contact or isolating themselves are classic manifestations of stress and pain in cats; coprostasis and anorexia may also be present during painful experiences. (c) Depression, reluctance to move, loss of appetite, and disinterest in surroundings are typical signs of pain in dogs. (d) Dogs with acute abdominal pain commonly adopt an unusual posture, known as the “prayer position”. Own elaboration, using information from Reid et al. [54]; Essner et al. [56].

- Cats. There are reports of the reduction of animal activity, appetite loss, tend to hide or evade interaction and may perform excessive licking of the affected area, interfering with their normal grooming (Figure 3(a, b) [54,56]). When cats experience severe pain, it is also possible to observe a rigid posture [57]. It is important to note that these behavioural modifications will be more evident if they are alone than when they are interacting with other individuals; in this case, their behaviour may be almost imperceptible or show no manifestations [58].
- Dogs. These animals tend to show exaggerated responses to harmful stimuli, being aggression a characteristic of acute pain, though it is more common to observe depression (Figure 3(c)), submission, anxious expressions, anorexia, licking of the affected zone, and a refusal to move. However, if the intensity of the pain is high, behaviours such as vocalizations, increased production of tears, constant touching of the affected area, wandering, and guardian-like behaviours may be observed [43,59]. Specific body postures are also identified in dogs with acute abdominal pain, known as the “prayer” position, which consists in raising their hindquarters and keeping their heads and front limbs on the floor (Figure 3(d)) [39,56].

### 2.3. Emotional level

Four aspects compose emotions; the first consists in the cognitive process that led to feelings; the second is biological and has an evolutive function; the third comprises physiological elements including, primarily, the autonomic nervous system and hormones; fourth and finally, there is the social component, which has a functional aspect that is purposeful and expressed through behaviour [60,61]. Therefore, an individual’s emotional state is the result of the interaction between the physiological activity and the cognitive evaluation of the situation [60], so that a mental state which leads to pain reflects a low level of welfare produced by the inability to achieve an adequate adaptation [62,63].

Also, research has determined that animals may show changes in the facial expression [64] due to harmful stimuli correlated with disturbances of behaviour and physiological parameters. However, tests conducted to date with dogs and cats have been inconclusive in terms of demonstrating their association with pain, as these responses can be altered by stress or some unwanted social interaction [43,62].

## 3. Scoring scales for evaluating pain

Quantifying pain is a difficult task because the physiological perception of this sensation is a multidimensional process. Thus, identifying pain in animals is complex due to the differences among species, behaviours and environments [65–67].

The literature consulted includes diverse scoring methods that consider both physiological and behavioural variables [67]. In veterinary medicine, the scoring methods used for pain in domestic animals are adapted forms of scales developed for humans [17]. The use of this scales has shown certain advantages in clinical veterinary practice, where the application and validation of an instrument designed to evaluate pain remarkably improve its management based on the scores obtained during assessment, working as an indicator of the level and and the treatment of analgesia required [68].

### 3.1. Subjective or unidimensional scales

If pain is considered only as a subjective experience, the valid measurement must attempt to accede to the subjective perception. The subjective criteria is the most significant limitation of this approach, since the observer must assign a degree according to her/his impressions and experience. For this reason, it is suggested that the same evaluator perform all procedures of pain assessment [54].

(a) Preventive scoring systems

Broadly-speaking, this system involves an evaluator feeling or perceiving a level of pain before the procedure regarding the “degree of pain” that the animal will experience after a given procedure. A score is assigned according to the level of pain that the animal is believed to experience. The categories are none, slight, moderate, or severe. The main disadvantage of this scale is that it does not determine the degree of pain in each patient individually; while its advantage is the simplicity to use and the possibility to plan a preventive analgesic strategy rapidly [39].

(b) Simple descriptive scales (SDS)

SDS consists in predefined categories or degrees of pain by assigning a number (value) to each category so that data can be managed statistically; for example 0, no pain; 1, medium level; 2, moderate level; 3, severe level. Some authors have used 4 or 5 categories. It is important to mention that the higher the number, the greater the difficulty in assigning the semiology of pain to each one. The disadvantages that such scales present are that they lack sensitivity when it comes to detect slight changes in the intensity of pain [19].

(c) Numerical rating scales (NRS)

It consists of a horizontal line divided into ten segments that are numbered sequentially from ‘0ʹ to ‘10ʹ. The value “0ʹ is equal to the absence of pain, while ”10ʹ indicates the worst pain. This scale does not permit decimal scores between the integers, so it is classified as a “discontinuous” scale that is subject to statistical error. Its advantage is that it takes into account various parameters, including physiological aspects, locomotor activity and vocalizations [19].

(d) Visual analogous scale (VAS)

These scales use a 100-mm long line with two endpoints: 0 mm (absence of pain) and 100 mm (maximum pain). The evaluator marks the point on the line that he/she considers correlates with the level of pain that the animal is experiencing. This scale equals the number obtained by measuring the distance from the initial point (0) to the spot where the mark was placed. The linearity of this method has been debated because it generates uncertainty as to whether or not there exists a proportional relationship between the values of different intensities. For example, a mark of 60 mm may not necessarily represent exactly double the amount of pain of a mark made at 30 mm [46].

Holton et al. [69] applied these three methods (AVS, SDS, NRS) to assess pain, finding that there is significant variability of 29–36% in results, depending on the observer. Given this problematic, Lascelles et al. [70] published an improvement of this scale that has been used in different studies [71,72]. Their version includes a dynamic and interactive assessment of patients (DIVAS), an additional component that entails observing the animal from a certain distance (DIVAS I); approaching the animal and interacting with it (DIVAS II); and, finally, auscultating the injured area (DIVAS III). When this pain assessment scale is applied, and the score for the animal is ≥40 mm, it is necessary to use rescue analgesia.

### 3.2. Objective or multidimensional scales

While subjective scales evaluate only one characteristic (behaviour or physiological constants), multidimensional scales consider both aspects. However, these scales also have a certain level of subjectivity, and their replicability is limited.

(a) Glasgow Composite Measuring Pain Scale (Glasgow Composite Measuring Pain Scale – CMPS)

Currently, this scale is validated to assess acute pain in dogs. It is a scoring system based on behaviour [1] that includes a structured questionnaire which is filled out by an observer following a standard protocol that covers evaluations of spontaneous behaviour, interactions with the animals, and clinical observations. This scale consists of seven assessment items: behaviour and reactions towards people, posture, mobility, activity, response to auscultation, treatment of the painful area, and vocalizations. For each one of these rubrics, there are specific questions that are selected by the observer according to the description that most closely reflects what is observed in the animal.

Although other composite scales have been designed, the CMPS is specific for veterinary science because it was designed using psychometric principles that generate and establish a procedure for selecting the relevant items. Finally, for this questionnaire to be used effectively in the dynamic environment of daily clinical practice, it must be short, simple and requires little time.

Reid et al. [68] published a simplified form of this scale (Short Form of the Glasgow Composite Measuring Pain Scale, SF-CMPS), which fulfils the characteristic of practicality, and specifies the score necessary to consider that an animal must receive analgesic treatment. The total score of this scale runs from 0–24 if the animal is mobile, but from 0–20 if mobility cannot be assessed. If the animal’s score is ≥6 on the former or ≥5 on the latter, it is necessary to use rescue analgesia.

(b) University of Melbourne Pain Scale

Originally developed as a multidimensional scale to evaluate pain in dogs by taking their behaviour and physiological constants as the basis of assessment [73], this scale considers six variables: physiological constants (cardiac and respiratory frequency, rectal temperature), response to auscultation, activity, emotional state, posture and vocalizations [74]. The evaluator assigns a value for each variable of the scale after observing the animal. This tool provides greater precision than the unimodal scales, and its specificity and sensitivity levels are higher because of the multiple factors considered. The disadvantage is that the typical animal behaviour must be known before it can be subjected to a surgical-anaesthetic procedure; hence, it is not a useful tool for sedated animals [45].

According to the score obtained on this evaluation, pain can be considered as slight (1–5 points), moderate (6–13), severe (14–21), or unbearable (21–27). It is necessary to use rescue analgesia when the animal score is ≥10.

(c) Colorado State University Feline and Canine Acute Pain

This scale is one of the few specific ones available for evaluating pain in both dogs and cats. It poses the most appropriate descriptions selected in the form of boxes that consider the following components: psychological/behavioural and response to auscultation. A third component is the rigidity of the body, which is assessed on a subjective scale called SDS. The scale includes schemes that help evaluators to identify the level of pain based on animal posture. Also, diagrams of different animal body positions are provided to help evaluators mark the zones or areas where the animal shows pain, tension or increased temperature [75].

(d) Botucatu University Pain Scale (UNESP-Botucatu-MCPS)

This is a scale designed with criteria that apply exclusively to cats. Its objective is to identify and quantify the intensity of pain in this species [57]. This scale considers the following variables: non-specific behaviours, response to auscultation of the surgical wound, reaction to auscultation of the abdomen/flank, vocalizations, posture, comfort, attitude, blood pressure, and appetite. The maximum value on this scale is 30 points. If a value ≥7 is obtained, it is necessary to apply analgesic treatment [58].

## 4. Monitoring perioperative pain

Detecting intraoperative nociception is the fundamental objective of the veterinary anaesthesiologist [14,76] since it is well-known that inadequate analgesia can lead to discomfort during recovery [10,11].

In this regard, assessing pain in anaesthetised animals is based on detecting haemodynamic reactivity, which is related to tachycardia and increased blood pressure, as well as changes in respiratory patterns or muscle tone. However, these modifications are not necessarily explicitly related to pain, and they can be influenced by the anaesthetic agents administrated. Therefore, we require a specific tool that can assess pain in animals under this condition and ensure early detection of haemodynamic reactivity. With this objective, a device based on the variability of the animal heart rate has been generated in veterinary medicine, having as a fundament the nociception-antinociception index by measuring the activity of the parasympathetic tone [14].

### 4.1. Parasympathetic tone activity index (PTA index)

A monitor for evaluating nociception in animals was introduced recently. This device derives from the analgesia/nociception index (ANI) used in children to detect intraoperative nociception [77,78]. The advantage is that it helps to assess acute pain not only in dogs and cats, but also in equine species.

This ANI index is produced by calculating the analysis of variability of the cardiac frequency (VCF) by taking into consideration the high-frequency component reflected in the R wave. This index is also influenced by the respiratory rhythm, which is included in the analysis. The ANI index is shown as a score from 0–100.

The way in which this index is obtained is recording a continuous ECG, detecting R waves, calculating the interval between waves (RR) and showing an average value of a reading made every 4 minutes, evaluating three types of waves according to the cause of the variation: high frequency (HF) waves (0.15–0.5Hz), manifest of parasympathetic tone activity; low frequency (LF) (0.004–0.15Hz), modulated by sympathetic tone; and very low frequency (VLF) (0.004–0.04Hz), related to thermoregulation and endocrine system [79,80].

The monitor PTA (PhysioDoloris®; Mdoloris Medical Systems, Lille, France) analyses the area below the curve of the HF component, a variable that, as mentioned, is exclusively related to the parasympathetic response and represents the influence of respiratory movements on the heart rate (HR), called respiratory sinus arrhythmia, it generates that the RH increases during inspiration in conjunction with a reduction in the RR interval and variability, while in the expiration the RH is reduced and the RR interval increases, increasing the variability due to a predominant parasympathetic tone [14,81].

The PTA index is calculated according to the following formula:

$$PTA=\left(100*\left[\alpha *AUCmin+\beta \right]/12.8\right)*100/161$$

where α = 5.1 and β = 1.2 have been determined in order to keep the coherence between the visual effect of the respiratory influence on RR intervals of the electrocardiogram and the quantitative measurement of ANI; 100/12.8 and 100/161 are coefficients determined to obtain PTA values between 0 and 100, with 100/161 being specific for the dog [14,82].

**Figure 4.**
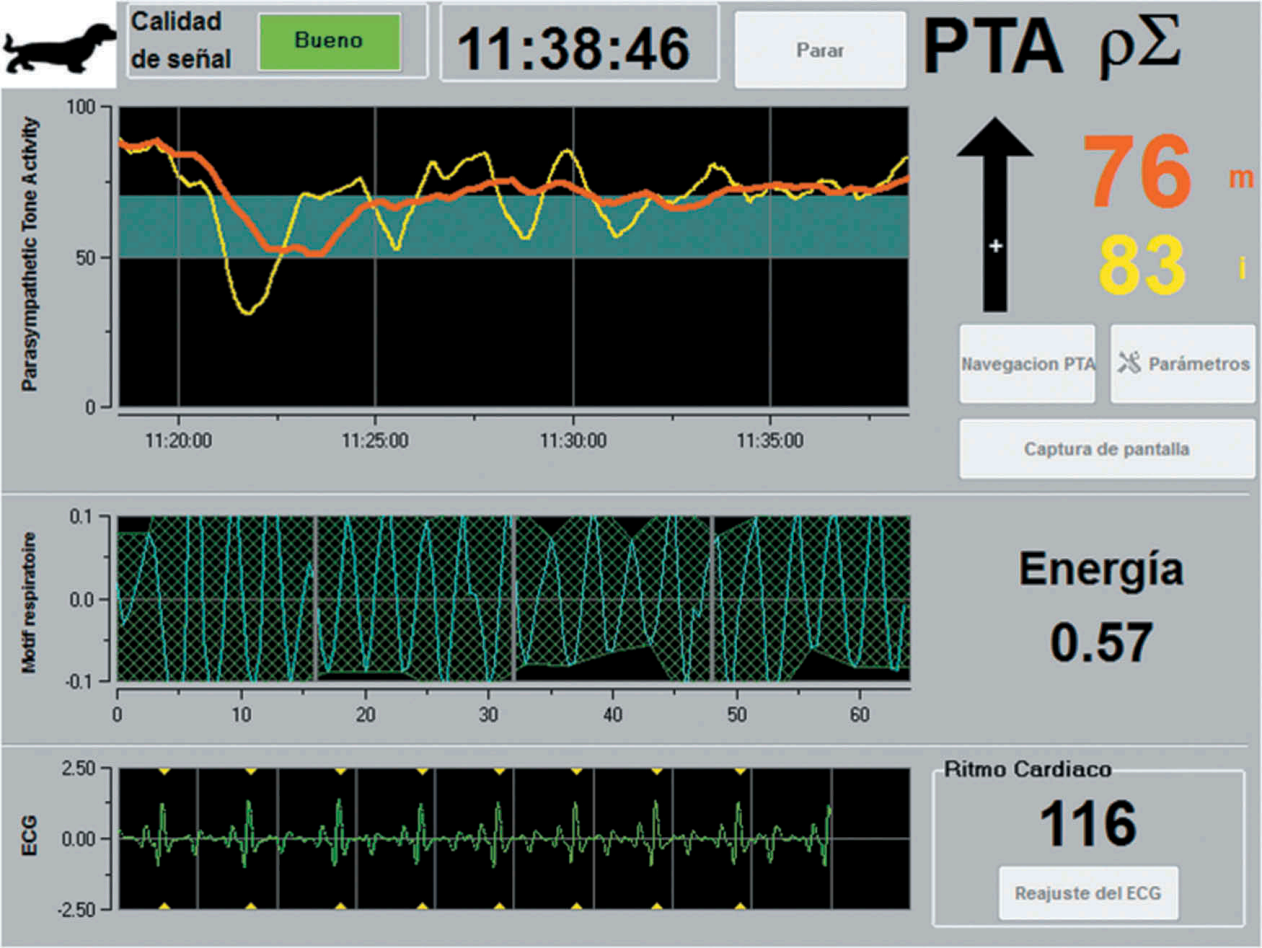
A PTA monitor, which uses the ECG signal to evaluate heart rate variability as a non-invasive method for assessing the autonomic nervous system. Recordings are characterized by two components: low frequencies (LF) (0.004–0.15Hz) influenced by the sympathetic system, and high frequencies (HF) (0.15–0.5Hz), which are related only to parasympathetic activity. In the image, the PTA interpretation concluded that the patient was not feeling pain. Own elaboration, using information from Aguado et al. [84].

Logier et al. [83] mention that this tool can show the balance of analgesia/nociception when evaluated during a nociceptive stimulus; where a high value of the parasympathetic tone reflects an absence of nociception, while a lower value reflects a potentially harmful stimulus (Figure 4 [84]).

**Figure 5.**
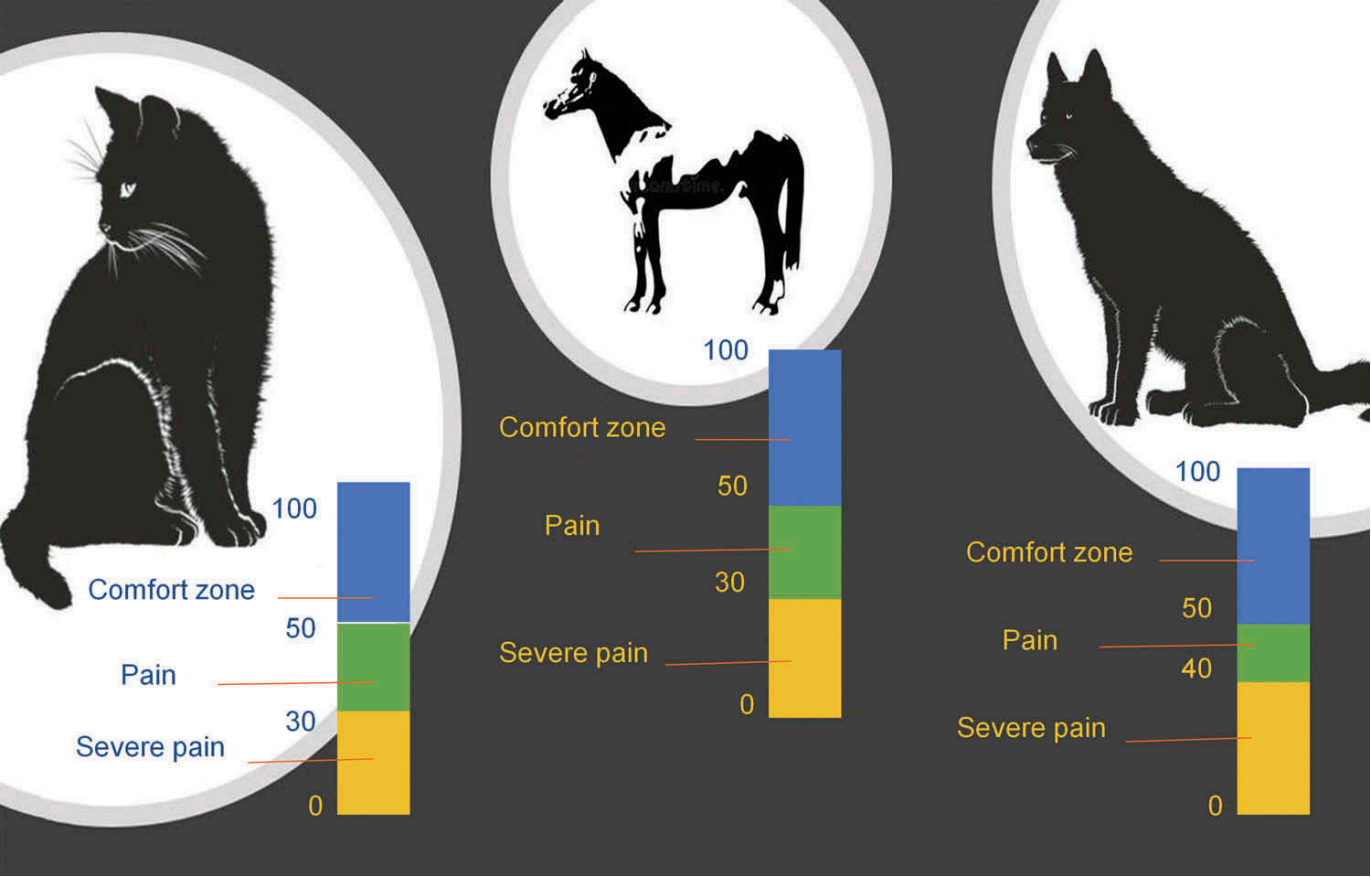
Clinical interpretation of PTA values in dogs, cats and horses. Own elaboration, using information from Mansour et al. [14].

Thus, based on different studies, this tool offers assistance in maintaining right analgesia/nociception balance, while making it possible to predict the haemodynamic reactivity of anaesthetized patients [14], considering values between 50–70 as ideal when the patient is under anaesthesia (Figure 5).

### 4.2. Bispectral index (BIS)

**Figure 6.**
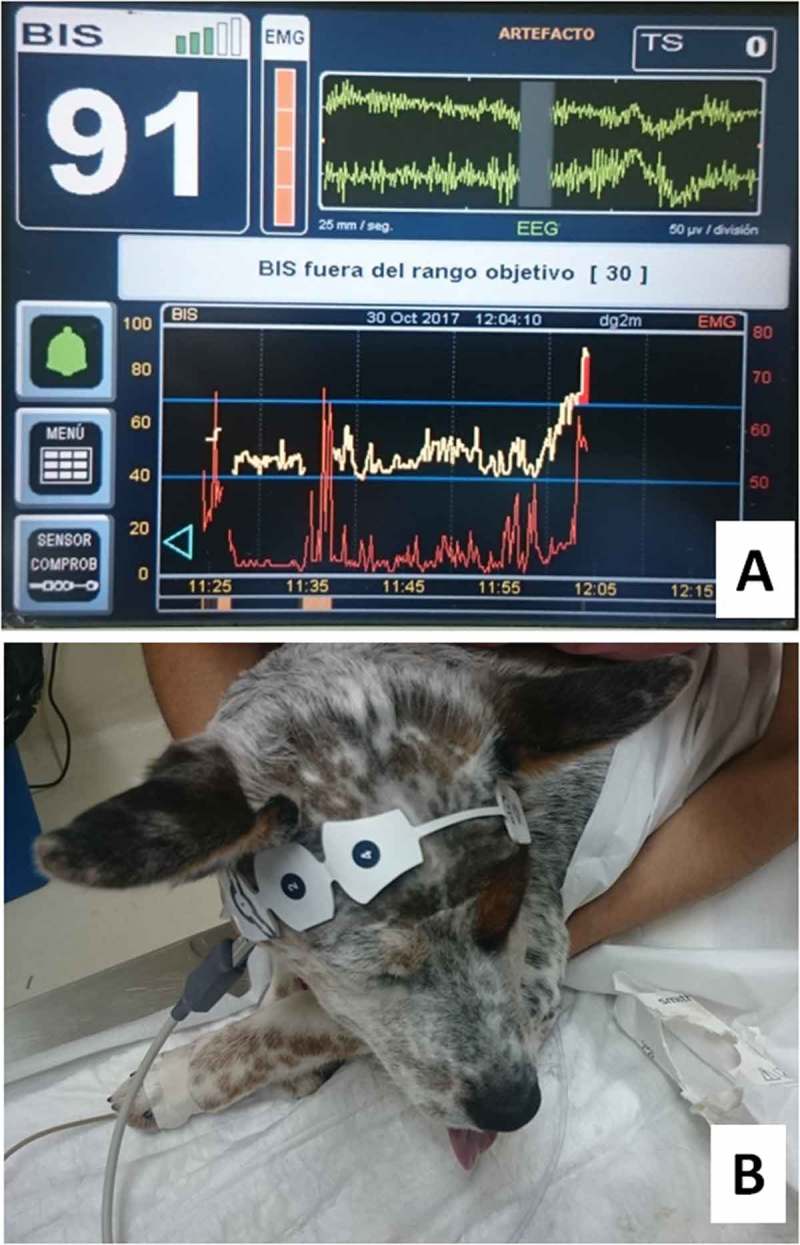
(a) BIS Monitor. In the image, a value of 91 indicates patient awareness after a surgical procedure, preventing an earlier extubating time. (b) BIS device consisting of an adhesive electrode sensor strip placed on the patient’s forehead. Own elaboration.

Anaesthesia is a state of decreased consciousness and sensation with varying degrees of muscular relaxation [85]. In veterinary practice, the tests traditionally used to assess nociceptive transmission during anaesthetic procedures are pinching a finger or the base of the tail and the evaluation of the palpebral and interdigital reflexes, besides of the mandibular tone. When the animal does not respond to such stimulus, it is inferred that it has achieved a state of unconsciousness [86]. Other parameters that have been used to know the level of consciousness and pain of an anaesthetic-surgical patient are the monitoring of haemodynamic changes (heart rate, blood pressure) and respiratory rate since sympathetic stimulation or lack of anaesthetic depth will generate tachycardia, hypertension and tachypnoea. However, these clinical signs of pain recognition and surgical stress may lack precision and sensitivity to identify it correctly [65,87]. In humans, a monitor has recently introduced for the intraoperative evaluation of consciousness and analgesia, called the bispectral index (BIS). It is based on an algorithm that depends on three factors of the specific linear and non-linear characteristics of an electroencephalogram (EEG), which correlate with the indexes of hypnosis. A linear parameter is generated with values of 0–100, which is proportional to the depth of the anaesthesia. This is interpreted as follows: the higher the BIS, the higher the level of consciousness of the evaluated patient. For example, a value of 100 is associated with an awake individual and the predominance of beta waves (captures above 13 Hz), while individuals with values of 90–80 present moderate sedation, and scores of 70–60 represent deep sedation (capturing alpha waves of 9–13 Hz). Values of 60–40 indicate that the organism is in optimal conditions for a sur-gical procedure because the predominant encephalographic waves are in the theta range (5–9 Hz) where recognition of pain is minimal (Figure 6(a, b)) [86].

Analgesia depends on decreasing the signs of ascending nociception, since inhibiting nociceptive transmission prevents the perception of a harmful stimulus; however, inadequate analgesia leads to the activation of brainstem structures which coactivate areas that induce cortical excitement [88,89]. It is important to consider that the capacity of response will depend not only on the level of analgesia and hypnosis, but also on the type and magnitude of the stimulus [86].

### 4.3. Other methods

In human medicine, other methods based on the analysis of the autonomic nervous system have been validated. They are supported by the use of sensors and algorithms that process physiological signals which permit the study of patients’ reactions to anaesthesia and surgery. These methods include pupillometry, surgical pleth index (SPI), skin conductance, CARDEAn (cardiovascular depth of analgesia), wavelet transform cardiorespiratory coherence, and photoplethysmographic waveform amplitude (PPGA) [77]. However, these methods have not yet been approved for veterinary medicine.

## 5. Conclusions

Nowadays, assessing pain has gained importance in animals and led to the design of more specialized systems to assess pain in different species [90–93]. The identification of pain suffered by patients has been a priority in medicine; however, it has become more important in veterinary medicine, because there are no comprehensive, sensitive and objective methodologies to assess the degree of pain that an animal perceives during perioperative procedures.

Due to their practicality, the Unidimensional or Multidimensional Scales are applied in veterinary clinics and hospitals to assess the degree of pain of animals under surgical procedure (pre and post); however several of them depend more than the evaluation criterion that of the degree of pain that the animal perceives.

Therefore, it is essential to consider the evaluation of PTA and BIS, under perioperative conditions, as objective evaluation alternatives for pain in veterinary medicine, in order to administer an adequate analgesic treatment.

PTA and BIS are integral, PTA and BIS are integral, and objective assessments that allow veterinarians – especially anaesthetists – to improve the management of pain in the patients.
